# Role of gut-brain axis in neurodevelopmental impairment of necrotizing enterocolitis

**DOI:** 10.3389/fnins.2023.1059552

**Published:** 2023-01-19

**Authors:** Yu Wang, Chengcheng Hang, Jun Hu, Chen Li, Canyang Zhan, Jiarong Pan, Tianming Yuan

**Affiliations:** ^1^Department of Neonatology, Children’s Hospital of Zhejiang University, Hangzhou, China; ^2^Department of Surgical Intensive Care Unit, Second Affiliated Hospital of Zhejiang University, Hangzhou, China

**Keywords:** necrotizing enterocolitis, neurodevelopmental impairment, gut-brain axis, microbiota, neurodevelopmental outcomes

## Abstract

Necrotizing enterocolitis (NEC) is a common gastrointestinal disease of preterm infants with high morbidity and mortality. In survivors of NEC, one of the leading causes of long-term morbidity is the development of severe neurocognitive injury. The exact pathogenesis of neurodevelopmental delay in NEC remains unknown, but microbiota is considered to have dramatic effects on the development and function of the host brain *via* the gut-brain axis. In this review, we discuss the characteristics of microbiota of NEC, the impaired neurological outcomes, and the role of the complex interplay between the intestinal microbiota and brain to influence neurodevelopment in NEC. The increasing knowledge of microbial-host interactions has the potential to generate novel therapies for manipulating brain development in the future.

## Introduction

Necrotizing enterocolitis (NEC) is an inflammation intestinal disorder, with variable damage to the intestinal tract including mucosal injury, necrosis, and intestinal perforation (Zani and Pierro, 2015). The typical neonate with NEC is a preterm infant who suddenly presents with feeding intolerance, abdominal distension, bloody stools and signs of sepsis (Neu and Walker, 2011). Despite improvements in neonatal intensive care, the mortality rates for NEC survivors remained 30–50% (Hall et al., 2013). NEC survivors often have life-long consequences, including neurodevelopmental delay, failure to thrive, gastrointestinal strictures and adhesions, cholestasis, short bowel syndrome, or intestinal failure (Bazacliu and Neu, 2019). Although the pathophysiology of NEC is not clear, it is likely to be multifactorial. Immaturity of the gut, intestinal ischemia, formula feeding and microbial dysbiosis contribute to inducing an uncontrolled inflammatory response in the gut and lead to the development of NEC. During the critical period of development, intestinal inflammation may adversely affect the maturation process of neurons and immune system (Strunk et al., 2014; Gussenhoven et al., 2018).

Recent evidence suggests a relation between intestinal microbiota, dysbiosis, and NEC (Niemarkt et al., 2015; Neu and Pammi, 2018). The occurrence of NEC is due to bacterial colonization in the immature host environment. It may be that the baseline response of intestinal mucosa of preterm infants to microbial ligands is enhanced, resulting in mucosal destruction and impaired mesenteric perfusion, increased expression of bacterial receptor Toll like receptor 4 (TLR4), and changes in other high response related factors. The recognition of NEC-specific dysbiosis can guide the prevention of NEC, timely diagnosis and targeted therapy. New technological advances provide more knowledge for the study of human microbiota and metabolic activities. The microbiota in newborns is of interest because it may be related to genetics, reproduction and the development of the immune system and brain.

Neurocognitive development is often impaired in patients with NEC. Epidemiological studies showed that 45% of NEC survivors suffered from a degree of neurodevelopmental delay such as cerebral palsy, hearing, visual, cognitive, and psychomotor impairments at 20 months of age (Rees et al., 2007; van Vliet et al., 2013). Severe neurocognitive impairment in preterm infants with NEC is more severe and persist than that in preterm infants without NEC (Shah et al., 2012; Wadhawan et al., 2014), suggesting a link between intestinal injury and toxicity to the brain of preterm infants. The exact mechanism of communication between the gut microbiota and the brain has not been elucidated yet. Moreover, NEC patients requiring surgery had worse mental and psychomotor developmental index scores around 2 years of age (Hintz et al., 2005)and various cognitive deficits at school age (Roze et al., 2011).

The severity of neuroinflammation is related to severity of NEC. The pro-inflammatory response in the brain leads to cell homeostasis and brain cell density changes in specific areas. These findings suggest that early intervention with NEC may reduce the incidence of acute neuroinflammation and brain injury.

The mechanisms underlying NEC-associated brain changes are largely unknown. The microbiota induced inflammatory cascade in NEC affects not only the intestine, but also the brain development through the so-called bidirectional gut-brain axis (Biouss et al., 2019). In this review, we review the current knowledge on microbiota in NEC, mechanisms of gut-brain axis, the neurocognitive consequences of NEC and explore the mechanisms of NEC related brain development impairments.

## Gut-brain axis

The gut-brain axis is a communication system that integrates the central nervous system (CNS), autonomic nervous system, the enteric nervous system, the hypothalamus - pituitary–adrenal axis (HPA), vagus nerve, intestinal microbiota, metabolic system and immunological signaling between the gut and the brain. It provides a potential route for intestinal microbiota and its metabolites to influence brain function. This communication system is bidirectional and enables the brain to influence gastrointestinal functions (such as motility, secretion and mucin production) and mucosal immune system cells which produced cytokines (Tracey, 2009).

Clinical observation found that orally administered antibiotics can reverse encephalopathy in patients with decompensated liver disease, this proves that commensal intestinal microbiota can influence brain function (Schiano, 2010). Studies reveal that the presence of intestinal microbiota in mice affects the development of neuronal circuitry involved in a wide range of activities, including memory, learning, motor control, and anxiety-like behavior (Gareau et al., 2011; Collins et al., 2012). Experimental studies demonstrated that intestinal microbiota in germfree mice (gnotobiotic mice) have the ability to influence brain development. Studies have shown that germ-free mice exhibit exaggerated stress-anxiety behavior with increased corticosteroids levels in response to mild stress and is normalized when germ-free mice were colonized with *Bifidobacterium longum* subsp. *Infantis* (Sudo et al., 2004).

Germ-free mice showed different levels of brain-derived neurotrophic factor, synaptophysin and synaptogenesis protein PDS-95, and neurotransmitters, such as noradrenalin, dopamine and serotonin, compared to colonized mice (Collins et al., 2012).

Probiotics have been shown to affect the brain through the vagus effect (Kunze et al., 2009; Bercik et al., 2010, 2011; Bravo et al., 2011). For example, a study showed that chronic administration of *Lactobacillus rhamnosus* activated exploratory behavior in mice and changes of GABA. This effect was not seen in vagotomized mice, indicating that the probiotic effects on the brain were vagal dependence (Bravo et al., 2011).

Bacterial metabolites have multiple effects on regulating the function of gut-brain axis, and microbiota is a major source of circulating organic acids and tryptophan metabolites (Wikoff et al., 2009). Short-chain fatty acids (SCFAs), which are produced by bacterial fermentation processes in the gut, include acetic, propionic and butyric acids. SCFAs are of importance in maintaining the integrity of the gut barrier and the blood-brain barrier (Diaz Heijtz, 2016). For example, high fecal concentrations of propionic acid correlate with anxiety in patients with irritable bowel disease (Tana et al., 2010) and may lead to abnormal behavior in children with autism spectrum disorder.

Tryptophan is metabolized through three metabolic pathways in the intestinal tract and balances with each other to maintain the homeostasis of intestinal immunity. The inflammatory stress response leads to the imbalance of metabolic pathways and the loss of intestinal immune homeostasis, which may produce neurotoxicity and lead to abnormal mental behavior.

Microbiota can produce multiple neurotransmitter compounds: serotonin, dopamine, norepinephrine, acetylcholine, and gamma-aminobutyric acid (GABA) (Lyte, 2011; Holzer and Farzi, 2014; Lu and Claud, 2019). 5-HT can not only regulate gastrointestinal peristalsis, secretion, vasodilation and nutrient absorption, but also regulate brain emotion, cognition and other functions. GABA is an important inhibitory neurotransmitter in CNS, has recently been shown to be produced by commensal *Lactobacilli* and *Bifidobacteria* (Barrett et al., 2012).

Endocrine cells located in the epithelial lining of the gut secrete hormones such as gastrin, cholecystokinin and 5-HT. The proximity of these cells to gut microbes raises the possibility of functional communication between gut microbes and gut endocrine cells. The microbiota can affect the number of intestinal endocrine cells and the release of active peptides (Uribe et al., 1994).

There is a delicate balance between the gut microbiota and the intestinal innate mucosal immune system under normal conditions. Changes in microbial composition caused by environmental factors, including diet, probiotics or antibiotics, disrupt this balance (Honda and Takeda, 2009). In NEC, LPS-containing microbes can induce inflammation in the brain by Toll-like receptor 4 (TLR4) activation-mediated cytokine release. The expression level of TLR4 in the intestinal tract of premature infants is higher than that in the full-term intestinal tract (Sodhi et al., 2010). Activation of TLR4 on the intestinal lining by gram-negative bacteria colonized in the intestinal tract of premature infants results in a number of harmful effects, including increased intestinal cell apoptosis, impaired mucosal healing, and enhanced release of pro-inflammatory cytokines, which overall contributes to the development of NEC (Lu et al., 2014).

In addition, factors involved in Toll-like receptor signaling include nuclear factor κB1, SIGIRR genetic variants (Sampath et al., 2015), myeloid differentiation-2 (MD-2) and GM2 activator protein (GM2A) genetic polymorphisms (Zhou W. et al., 2015), IL-4ra mutant allele which associated with immune phenotype shift from type 1 to type 2 T helper cells (Treszl et al., 2003). These gene expressions could all influence the development of NEC.

Central nervous system disorders also influence the composition of gut microbes. Stress-induced changes in intestinal motility and mucus secretion lead to changes in microbial habitat (Collins et al., 2012). Stress also increases the concentration of norepinephrine in the gut, which may lead to changes in microbial composition during stress.

## Microbiota and NEC

Necrotizing enterocolitis is associated with inappropriate intestinal colonization in preterm infants. Experimental models showed that NEC occurs in conventionally raised animals but not in germ-free animals (Lawrence et al., 1982; Afrazi et al., 2011).

Following delivery, the gut becomes rapidly colonized with commensal bacteria derived from maternal colonic and vaginal flora (Patel and Denning, 2015), and from the surrounding environment. These commensal bacteria act on the immune system and the metabolism of microorganism, maintain intestinal homeostasis, and protect the gut from injury (Rakoff-Nahoum et al., 2004; Tokarz-Deptula et al., 2016). A study showed that gut colonization of the premature infants took place in an orderly process, from *Bacilli* to *Gammaproteobacteria* to *Clostridia* (La Rosa et al., 2014). Exogenous factors such as antibiotics, delivery mode, feeding and age could influence the pace, but not the “predestined” sequence, of progression. Compared with full-term infants, premature infants have lower microbial diversity, lower presence of intestinal commensal microbes (such as *B. longum* species and *Bacteroidetes*) and higher numbers of potential pathogenic bacteria *Clostridiaceae* and *Enterobacteriaceae* (Vongbhavit and Underwood, 2016; Wandro et al., 2018). The rate of microbial populations aggregate depends on gestational age. The more premature the infant was, the slower the process of bacterial colonization was. Several factors play a role in the development of the gut microbiota after delivery such as antibiotics, probiotic supplementation, types of infant feeding, gestational age and infant hospitalization (Penders et al., 2006; Madan et al., 2012; Raba et al., 2021). These factors influence the composition of the microbiota measured at 1 month of full term, and remained different at 4 months of age but converged at 1 year of age (Azad et al., 2013).

Overall, this dysbiosis in NEC was characterized by increased abundances of *Proteobacteria* and low abundances of *Firmicutes* and *Bacteroidetes* (Pammi et al., 2017; Olm et al., 2019). Studies provide further evidence that the microbiome shifts prior to the onset of NEC (Sim et al., 2015). A study revealed that there were more *Klebsiella*, bacteria encoding *fimbriae*, and bacteria encoding for gene clusters of secondary metabolites associated with quorum sensing and bacteriocin production (Olm et al., 2019). Bacterial replication rates are significantly higher before NEC development especially *Enterobacteriaceae*.

Some studies also found that the specific microbiota profile differ depending on the age of disease onset. NEC preceded by *Firmicutes* dysbiosis (increased abundances of *Firmicutes* and accompanied by decreased *Gammaproteobacteria*) occurred earlier (onset, days 7 to 21) than NEC preceded by *Proteobacteria* dysbiosis (increase in *Gammaproteobacteria* and accompanied by decrease in *Firmicutes*) (onset, days 19 to 39) (Morrow et al., 2013; Zhou W. et al., 2015).

Antibiotic therapy has been suggested to increased risk of NEC in preterm infants by reducing the diversity of microbiota, which might lead to pathogenic microbes overgrow over the commensal species. The relative abundance of *Proteobacteria* increased and *Firmicutes* and *Actinobacteria* decreased in infants treated with antibiotics (Pammi et al., 2017).

The mode of delivery is the most important factor affecting the composition of microbiota. *Bifidobacterium*, *Escherichia* (*Escherichia coli*), *Bacteroides* and *Parabacteroides* were higher after vaginal delivery (Shao et al., 2019). By contrast, the relative abundance of *Firmicutes* (*Enterococcus faecalis, Enterococcus faecium, Staphylococcus epidermis, Streptococcus parasanguinis*, and *Clostridium perfringens*) and *Klebsiella oxytoca, Klebsiella pneumoniae, Enterobacter cloacae* were higher after Caesarean section (Shao et al., 2019). But it is not clear whether this has anything to do with NEC’s development.

Feeding style affects the composition of intestinal microbiota. Formula-fed infants showed a higher abundance of *Firmicutes*, and breast-fed infants showed higher relative abundances of *Proteobacteria*, but its association with NEC development is not for sure (Pammi et al., 2017). Breast milk contains certain macronutrients, polyunsaturated fatty acids, lactoferrin, immune cells, and immunoglobulins and growth factors. An outstanding research reports that IgA in maternal milk shapes the host–microbiota relationship of preterm neonates and that maternal IgA is a critical for the prevention of NEC (Gopalakrishna et al., 2019).

Probiotics may reduce the risk of necrotizing necrosis by regulating the gut microbiome. But its effect is not entirely determined. A Cochrane Database in Sharif et al. (2020) reviewed probiotics in very preterm or very low birth weight infants to prevent necrotizing enterocolitis. Meta-analysis showed that probiotics may reduce the risk of NEC: RR 0.54, 95% CI 0.45 to 0.65 (54 trials, 10,604 infants). The effects of probiotics of different subgenus species on the reduction of NEC and nervous system in preterm infants are shown in Table 1. However, the certainty of the evidence is low because of the limitations of trial design (most trials are small in size) and funnel plot asymmetry consistent with publication bias. Large-scale, high-quality trials are therefore needed. Another meta-analysis (Morgan et al., 2020) exploited that among interventions compared with placebo, the combinations of *Lactobacillus* spp and *Bifidobacterium* spp (OR 0.35, 95% CI 0.20 to 0.59), *Bifidobacterium animalis* subsp, *lactis* (OR 0.31, 95% CI 0.13 to 0.74), *Lactobacillus reuteri* (OR 0.55, 95% CI 0.34 to 0.91), or *L. rhamnosus* (OR 0.44, 95% CI 0.21 to 0.90) significantly reduced severe NEC. Although probiotics may reduce the risk of NEC, their safety or long-term efficacy has not been adequately studied in premature infants.

**TABLE 1:** Probiotics for very preterm or very low birth weight infants to prevent Necrotizing enterocolitis (NEC) based on Cochrane Database.

Probiotics versus control, subgroup			
	No. of participants	Effect size (risk ratio M-H, Fixed, 95% CI)*	References
***Outcome 1:***			
*Necrotizing enterocolitis*	10,604	0.54 (0.45, 0.65)	
*Bifidobacterium* spp. Plus *Lactobacillus* spp.	2,041	0.36 (0.23, 0.59)	Lin et al., 2005, 2008; Rouge et al., 2009; Samanta et al., 2009; Braga et al., 2011; Al-Hosni et al., 2012; Roy et al., 2014; Saengtawesin et al., 2014; Van Niekerk et al., 2014; Chowdhury et al., 2016; Strus et al., 2018
*Bifidobacterium* spp.	2,988	0.72 (0.54, 0.96)	Kitajima et al., 1997; Fujii et al., 2006; Mohan et al., 2006; Stratiki et al., 2007; Wang et al., 2007; Mihatsch et al., 2010; Dilli et al., 2015; Costeloe et al., 2016; Hays et al., 2016; Totsu et al., 2018; Oshiro et al., 2019; Agrawal et al., 2020
*Lactobacillus* spp.	2,000	0.45 (0.28, 0.71)	Reuman et al., 1986; Millar et al., 1993; Dani et al., 2002; Manzoni et al., 2006, 2009; Chrzanowska-Liszewska et al., 2012; Akar et al., 2017; Indrio et al., 2017; Wejryd et al., 2019
*Bifidobacterium* spp. Plus *Lactobacillus* spp. Plus *Streptococcus* spp.	662	0.42 (0.22, 0.77)	Fernandez-Carrocera et al., 2013; Kanic et al., 2015
*Bifidobacterium* spp. Plus *Streptococcus* spp.	1,244	0.36 (0.19, 0.68)	Bin-Nun et al., 2005; Jacobs et al., 2013
*Saccharomyces* spp	621	0.82 (0.44, 1.50)	Costalos et al., 2003; Demirel et al., 2013; Serce et al., 2013; Zeber-Lubecka et al., 2016
*Bifidobacterium* spp. Plus *Lactobacillus* spp. Plus *Saccharomyces* spp	583	0.67 (0.28, 1.58)	Dutta et al., 2015; Shashidhar et al., 2017
*Bacillus* spp.	465	0.61 (0.23, 1.61)	Sari et al., 2011; Tewari et al., 2015
***Outcome 2:***			
*Severe neurodevelopmental impairment*	1,518	1.03 (0.84, 1.26)	
*Bifidobacterium* spp. Plus *Lactobacillus* spp.	269	1.27 (0.81, 1.98)	Chou et al., 2010
*Bifidobacterium* spp.	162	0.77 (0.34, 1.72)	Totsu et al., 2018
*Lactobacillus* spp.	249	1.01 (0.69, 1.48)	Akar et al., 2017
*Bacillus* spp.	174	1.09 (0.58, 2.07)	Sari et al., 2011
*Bifidobacterium* spp. Plus *Streptococcus* spp.	664	0.97 (0.69, 1.36)	Jacobs et al., 2013

*Effect size is based on Cochrane Database of systematic reviews (Sharif et al., 2020).

## Neurodevelopmental outcomes in survivors of NEC

Premature infants with NEC have a high incidence of morbidity and mortality. Complications include gastrointestinal problems (including strictures and adhesions, cholestasis, motility disturbances, short bowel syndrome, intestinal failure, feeding difficulties), poor growth and more important worse neurodevelopment outcome [intraventricular hemorrhage (IVH), periventricular leukomalacia (PVL) and white matter injury, predictive of long-term neurodevelopmental impairment]. There is still a possibility of extended hospitalization, repeated surgeries, closer follow-up, and treatment of long-term complications of NEC (Hayakawa et al., 2015; Bazacliu and Neu, 2019).

Necrotizing enterocolitis has a substantial impact on motor, cognitive and behavioral performance outcomes in early childhood later in life (Waugh et al., 1996; Chacko et al., 1999; Sonntag et al., 2000; Castro et al., 2004; Salhab et al., 2004; Hintz et al., 2005; Schulzke et al., 2007; Lodha et al., 2010; Pike et al., 2012; Shah et al., 2012; Zozaya et al., 2021). The association between NEC and neurocognitive impairment has been confirmed by many researchers. Schulzke et al. (2007) analyzed eleven studies and revealed a strong correlation between neurodevelopmental delay and children with stage II or higher NEC, especially if they require surgery for the illness. Another systematic review by Rees et al. (2007) included data from 7,843 preterm infants (821 with NEC) from ten studies found significantly worse neurodevelopmental outcomes in NEC compared with than that of prematurity alone. Characteristics of neurodevelopmental impairment (NDI) related studies on NEC are summarized in Table 2.

**TABLE 2:** Neurodevelopmental impairment (NDI) studies of Necrotizing enterocolitis (NEC) among preterm infants.

References	Centers	Assessment tool	Birth weight (g) [mean (range) of NEC]	Duration of follow up (months corrected age)	Number with NEC attended follow-up (*n*)	Total number of attended follow-up (*n*)	Definitions of NDI
Shah et al., 2012	Multi	BSID II	ELBW	Hospital discharge, 18 and 22 months	62	785	NDI: at least include 1 of the following: MDI < 70, PDI < 70, CP, hearing impairment, visual impairment
Hintz et al., 2005	Multi	BSID II	ELBW	18,22	245	2,703	NDI: at least include 1 of the following: MDI < 70, PDI < 70, CP, blindness, deafness.
Zozaya et al., 2021	Multi	BSID-III	ELBW	18–30	176	2,019	NDI:CP with GMFCS score, Bayley-III component score of < 85, hearing loss, or visual impairment
Hansen et al., 2019	Single	Strength and Difficulties score	1442.5	126	163	400	Borderline/abnormal SDQ-score; CP
Wadhawan et al., 2014	Multi	BSID-II; Amiel-Tison assessment	ELBW	18–22	472	8,938	moderate or severe cerebral palsy, bilateral blindness, bilateral hearing loss needing amplification, MDI or PDI less than 70
Lodha et al., 2010	Two	BSID-II	1,145	24–28	10	40	NDI: Includes MDI < 70, PDI < 70, and CP.
Dilli et al., 2012	Single	BSID-II	VLBW	18–24	20	60	moderate-to-severe cerebral palsy; MDI or PDI < 70; bilateral deafness; or bilateral blindness.
Sonntag et al., 2000	Single	Griffiths	VLBW	12,20	20	60	NDI: GQ < 2 SDs below mean
Salhab et al., 2004	Single	BSID-II	ELBW	18–22	17	68	No clear definition. Data on mean MDI and PDI given
Soraisham et al., 2006	Single	BSID-II and Stanford- Binet	BW below 1,250 g	36	46	146	NDI: at least include 1 of the following: cognitive delay (MDI or IQ < 2 SDs below mean), CP, visual impairment, deafness.
Roze et al., 2011	Single	Movement-ABC; WISC-III-NL; Child Behavior Checklist	1,020 –1,775	6–13 Year	32	83	Data on mean Motor outcome, Cognitive outcome and Behavioral outcome are given
Allendorf et al., 2018	Single	BSID-II	1,039	24	37	76	No clear definition. Data on mean MDI and PDI given
Martin et al., 2010	Multi	BSID-II	n/a, 23–27 weeks gestation	24	101	1,155	moderate-to-severe cerebral palsy; MDI or PDI < 70;
Fullerton et al., 2017	Multi	BSID-II or BSID-III	ELBW	18–24	866	9,929	Severe neurodevelopmental disability: bilateral blindness, hearing impairment requiring amplification, inability to walk 10 steps with support, cerebral palsy, and/or Bayley Mental or Psychomotor Developmental Index < 70.
Pike et al., 2012	Multi	Health Utilities Index (HUI)and Strength and Difficulties score	n/a	7Y	119	6,615	Based on HUI-3 classification
Castro et al., 2004	Multi	BSID-II	ELBW	18–22	72	1,151	NDI: Includes MDI < 70, PDI < 70, and CP.
Chacko et al., 1999	Single	Griffiths and Stanford-Binet	ELBW	12–59	40	60	NDI: at least include 1 of the following: GQ or IQ < 71, moderate or severe CP, blindness, deafness
Waugh et al., 1996	Single	Griffiths and McCarthy Index	ELBW	24	23	198	NDI: GQ < 76 (2 SDs below mean), CP, Visual impairment, Deafness
Tobiansky et al., 1995	Single	Griffiths and Stanford-Binet	VLBW	36–60	49	89	NDI: at least include 1 of the following: GQ or IQ < 71, moderate or severe CP, blindness, deafness
Mayr et al., 1994	Single	Denver test, drawing test	VLBW	12–156	12	18	No clear definition
Simon et al., 1993	Single	BSID I	VLBW	8,15,24	18	18	No clear definition. Data on mean MDI and PDI given
Ganapathy et al., 2013	Multi	n/a	All neonates	6, 12, 24,36	250	2,899	No clear definition
Hayakawa et al., 2015	Multi	n/a	VLBW	18	44	394	NDI was defined as the presence of neurological sequelae in infants at 18 months of corrected age.

NDI, neurodevelopmental impairment; n/a, data is not available in respective studies; BSID, Bayley Scales of Infant and Toddler Development; WISC-III-NL, Wechsler Intelligence Scale for Children, third edition, Dutch version.

Several small matched control studies revealed a significant correlation between NEC and brain injury (Simon et al., 1993; Mayr et al., 1994; Tobiansky et al., 1995; Ganapathy et al., 2013; Allendorf et al., 2018; Hansen et al., 2019). A recent study showed that stage II or III NEC infants had a significantly higher risk of cognitive delay and visual impairment compared to age-matched controls at 36 months (Soraisham et al., 2006). Another study found a significant neurodevelopmental delay at 12 and 20 months in NEC infants compared to age-matched controls (Sonntag et al., 2000). Very low birth-weight babies with NEC had increased risk of severe head growth failure (Regev et al., 2016). Moreover, recent studies demonstrated that NEC was correlated with lower psychomotor developmental index (PDI) scores (Salhab et al., 2004; Dilli et al., 2012) and mental developmental index (MDI) (Dilli et al., 2012). The evidence for their impaired performance in cognitive and developmental assessments is supported by Bayley Scales of Infant Development, the Griffiths Quotient and the Stanford–Binet test. Studies using brain magnetic resonance imaging (MRI) showed that white matter and cortical abnormalities in infants with NEC (Shah et al., 2008; Lee et al., 2014; Merhar et al., 2014; Shin et al., 2016).

The presence of advanced NEC or need for surgery increase the risk of neurological impairment. A large retrospective analysis showed that surgical NEC but not medically managed NEC was a significant independent risk factor for worse mental and psychomotor developmental index scores at 18–22 months corrected age compared with control (Hintz et al., 2005). Several studies indicate that children with surgical NEC have poorer neurodevelopmental outcomes (Rees et al., 2007; Schulzke et al., 2007; Martin et al., 2010; Fullerton et al., 2017) and more brain injury on MRI compared to medical infants (Merhar et al., 2014). But not all the data fully support this conclusion (Dilli et al., 2012). Shah’s team found an increased risk of neurodevelopmental disorders measured by Bayley test in ELBW infants with NEC and spontaneous intestinal perforation (SIP), and there were no significant differences in neurodevelopmental outcomes between NEC medical and surgical groups observed in the study (Shah et al., 2012).

Experimental NEC mice study further explored the morphologic changes in the brain. NEC pups had smaller brain weight and a thinner cerebral cortex compared to the control group (Biouss et al., 2019). In particular, in specific regions of the NEC brain, the number of neurons, oligodendrocytes, and neural progenitors were reduced. There is an impairment in neurogenesis and loss of myelin (Nino et al., 2018; Zhou et al., 2021) which may lead to poor performance in adult life. Levels of apoptosis and ER stress were increased in NEC. In addition, pro-inflammatory cytokines (IL-6 and TNFα) and the density of activated microglia and astrocytes were increased in the brain, which were positively correlated with the increase of intestinal proinflammatory cytokines and the severity of NEC injury, respectively (Biouss et al., 2019).

Necrotizing enterocolitis is often associated with sepsis, which itself is associated with adverse neurocognitive outcomes (Salhab et al., 2004; Soraisham et al., 2006; Martin et al., 2010). Other preterm complications associated with NEC may also conspire to affect brain development.

## Proposed mechanisms underlying associations between NEC and neurodevelopment

Early life is critical period of rapid brain development, including neurogenesis, neuronal migration, maturation, apoptosis and synaptogenesis (Hickey et al., 2018). These processes are also influenced by several other factors, such as malnutrition, hypoxia and ischemia, and the inflammatory cytotoxic mediators. The development of NEC at a critical stage of brain development can cause neurodevelopmental disorders and their association is multifactorial (Keunen et al., 2015; Hickey et al., 2018; Niemarkt et al., 2019).

Necrotizing enterocolitis intestinal injury send intestinal microbiological signals associated with systemic inflammation transmitted to the brain and limbic system *via* enteric nervous system, autonomic nervous system, and hypothalamic-pituitary axis Moschopoulos et al. (2018) suggest that primary laparotomy rather than primary peritoneal drainage should be used in children with advanced NEC, as the continued damaging of residual necrotic intestine is harmful to the neonatal brain.

**FIGURE 1:**
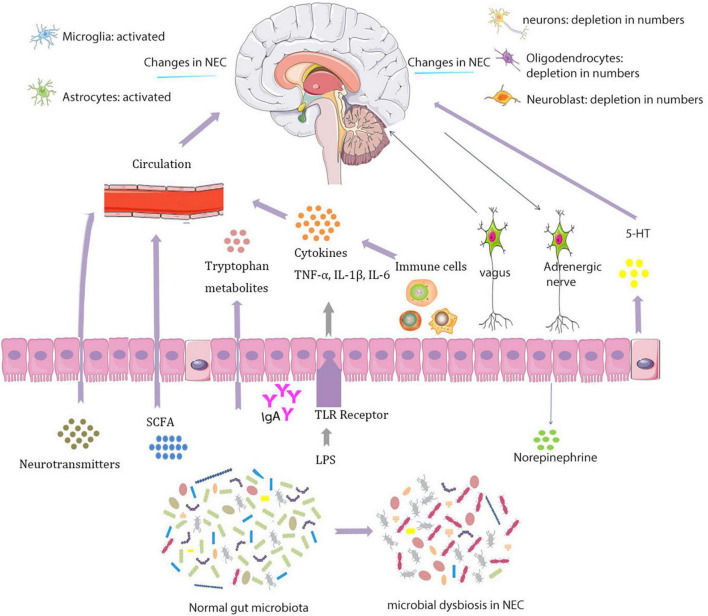
Interactions between the microbiota and nervous systems in necrotizing enterocolitis (NEC).

In experimental NEC brain, there are fewer neurons, oligodendrocytes, and neural progenitors that may affect neurological function in specific regions of the brain (Biouss et al., 2019) (Figure 1). The study found that in NEC’s piglet model, a series of hippocampal differentially expressed genes related to neuroinflammation and hypoxia are increased and expressions of genes related to protection against oxidative stress and oligodendrocytes are downregulated (Sun et al., 2018).

Studies found that NEC-associated brain injury is characterized by microglial activation, loss of white matter and cognitive dysfunction, the mechanism of which remains unclear (Nino et al., 2018). The underlying mechanisms of white matter injury may include cerebral ischemia, systemic infection or inflammation, and the inherent vulnerability of premyelinated white matter oligodendrocytes. Premyelinating oligodendrocytes are very vulnerable to ischemia and inflammation in the process of encephalopathy (Volpe, 2011). White matter injury is often manifested as periventricular leucomalacia (PVL) (Hintz et al., 2015), which is sign of disrupted brain development and neurological dysfunction in premature babies. Neuronal dysfunction may be the result of excessive cell degeneration or lack of neurogenesis. Neuronal degeneration is in part by exposure to factors predisposed to NEC, such as hypoxic ischemic injury, which increases cell apoptosis and promotes inflammatory response.

Intestinal injury and barrier dysfunction provide bacteria and inflammatory mediators a potential route to transfer to the systemic circulation, leading to a systemic inflammatory response. Cytokines produced during this process disrupt preterm brain development (Lodha et al., 2010; Hickey et al., 2018). Levels of pro-inflammatory cytokines and activated microglia and astrocytes density are increased in NEC-associated brain injury (Zhou et al., 2021). Cytokines have dramatic effects on the development of neurogenesis, neuronal migration, synaptogenesis, and synaptic plasticity (Williamson et al., 2011; Bilbo and Schwarz, 2012; Donzis and Tronson, 2014). Studies serve to illustrate that early life exposure to lipopolysaccharide (LPS) results in dysregulated neurogenesis, increased microglia, reduced hippocampal volume, axonal injury and memory impairment in rats (Bilbo et al., 2005; Wang et al., 2013; Smith et al., 2014; Sun et al., 2018).

The response of TLR4 to LPS of gut bacteria (most often *Gammaproteobacteria*) is exaggerated in NEC model (Hackam et al., 2019; Niemarkt et al., 2019), resulting in signaling cascades and activated nuclear translocation of nuclear factor kappa-β (NFκB). NFκB signaling pathway is involved in plasticity and survival in neurons, and mediates pro-inflammatory responses in glial cells (Crampton and O’Keeffe, 2013). TLR4 is of critical importance in NEC. NEC is not induced in TLR mutants or knockout animals. Clinical trials have hinted that probiotics activate TLR9 and in turn limit TLR4, thereby relieving NEC (Hackam and Sodhi, 2018; Hackam et al., 2019).

Activated microglia has a central role in white matter injury in newborn mice (Tahraoui et al., 2001). Changes in the early stage of acute inflammation may be related to TLR4-mediated microglia activation (Liddelow et al., 2017; Nino et al., 2018). Activated microglia induce an increase in astrocyte numbers in the late stages of inflammation, a process known as reactive astrocytosis (Liddelow et al., 2017; Sochocka et al., 2017). Neuroinflammatory astrocytes could also release toxic factors that can lead to progressive neuronal loss (Bi et al., 2013; Liddelow et al., 2017; Biouss et al., 2019). TLR4-dependent microglia and astrocytes are activated in the brain of NEC. The excessive astrocyte and microglia activation has been shown to lead to a loss of oligodendrocyte progenitor cells in response to TNFα exposure (Jurewicz et al., 2005; Nino et al., 2018; Biouss et al., 2019) and exacerbate white matter and periventricular injury (Billiards et al., 2006; Selip et al., 2012).

Furthermore, intracerebroventricular injection of NEC enteric-derived CD4 + T lymphocytes into Rag1-/- recipient mice lacking CD4 + T cells induced brain injury, suggesting that enteric-derived T lymphocytes may mediate neuroinflammation of NEC. These findings indicate that enterogenic IFN-γ- releasing CD4 + T cells may induce NEC-related brain injury. This was confirmed in human specimens, where the brains of infants with NEC were examined and compared with those of the control group, exhibiting an increased accumulation of CD4 + T lymphocytes (Zhou et al., 2021).

Microbiota and brain-gut axis are crucial for NEC brain development. The role of the intestinal microbiota in the prevention, pathogenesis and potential neurodevelopmental consequences of NEC are necessary to review here (Hickey et al., 2018). The mechanisms underlying microbiota on brain functioning are several.

First, microbiota cause and may exacerbate systemic inflammation. In addition, dysbiosis may result in reduced intestinal and blood-brain barrier function *via* SCFA production (Diaz Heijtz et al., 2011; Lu et al., 2018). SCFAs have been shown to influence barrier function of the intestinal epithelial cells. Dysbiosis may disturb the content difference of SCFA in the gut (Neu and Pammi, 2018). Both high and low SCFA levels were associated with NEC (Lin, 2004). SCFA and bile acids affect the production of serotonin, which in turn regulates intestinal motility (Niemarkt et al., 2019). Intestinal microbial metabolites (mainly SCFA) prevent microglial activation and subsequent neuroinflammation through immune modulation (Erny et al., 2015).

Besides, microbiota may influence the brain through the production of neurotransmitters (serotonin, melatonin, GABA, dopamine) or by stimulating intestinal cells to do so. Gut microbes transmit signals to the brain *via* the vagus nervous system, and vice versa. Although it is known that probiotics may mitigate the effect of dysbiosis and further improve brain development, more research is needed into the relationship between NEC microbiota and brain development in preterm infants.

Necrotizing enterocolitis is often accompanied by malnutrition, which can lead to poor brain development. Children with NEC not only had insufficient nutritional intake, but also sustained mucosal injury resulted in reduced nutritional absorption. Studies have shown that high protein provision in infants with surgical NEC was associated with increased head circumference, an index of brain growth (Lin et al., 2017). Protein status is important due to its role in fat-free mass (FFM) accretion, neurogenesis, neuronal differentiation and involved in cognition related insulin-like growth factor-1 (IGF-1) expression (Hansen-Pupp et al., 2013; Pfister et al., 2013). Micronutrients iron and zinc deficiency also contribute to abnormal neurodevelopment and poor growth (Ramel et al., 2014).

## Conclusion

Premature infants with NEC have poor neurodevelopmental outcomes, but the underlying pathogenic mechanism has not been clearly established and it is likely to be multifactorial. Microglial proliferation, astrocyte proliferation, decreased neurogenesis and demyelination are important characteristics of NEC brain changes. Gut microbiota alterations contribute to the pathogenesis of NEC. Dysbiosis causes changes in the brain through TLR4-mediated inflammation, SCFA and neurotransmitters. The recognition of NEC-specific gut-brain axis can guide the prevention of NEC, timely diagnosis and targeted therapy. Studies on how NEC causes neurological impairment and its therapeutics are few. There is ample room for pathophysiological mechanism and clinical application research aimed at improving the prognosis of NEC.

Biomarkers predicted by microbiome and metabolomics are promising tools for accurate and timely prediction of NEC and selection of appropriate treatments. More studies with longer follow-up periods are needed to better understand the brain development of these patients. Further studies are needed to determine the most effective type of probiotics, dosage, administration time, and duration to reduce the incidence of NEC.

## Author contributions

All authors listed have made a substantial, direct, and intellectual contribution to the work, and approved it for publication.
